# COVID‐19 vaccine wastage: A challenge to achieving herd immunity in Nigeria

**DOI:** 10.1002/puh2.55

**Published:** 2023-01-11

**Authors:** Shuaibu Saidu Musa, Adamu Muhammad Ibrahim, Muhammad Rahima Hamid, Hope Jonah Obia, Garba Mohammed Buwa

**Affiliations:** ^1^ Department of Nursing Science Ahmadu Bello University Zaria Nigeria; ^2^ Global Health Focus Africa Abuja Nigeria; ^3^ Department of Immunology Usmanu Danfodiyo University Sokoto Nigeria; ^4^ Department of Chemistry Abubakar Tafawa Balewa University Bauchi Nigeria; ^5^ Department of Public Health University of Calabar Calabar Nigeria; ^6^ State Specialist Hospital Gombe Nigeria

## Abstract

The emergence of the COVID‐19 pandemic has overburdened many health‐care systems in the world, particularly in Nigeria and other African countries with fragile public health system. Since the emergence of the pandemic, Nigeria has been struggling to restore the impact on its health‐care system as the country keeps recording a growing number of cases and deaths. Vaccination has been the most efficient strategy towards curbing the COVID‐19 pandemic, particularly in countries like Nigeria where preventive measures were poorly strategised or implemented. The country was able to secure some doses of the COVID‐19 vaccine through donations, but concerns have been raised as up to 1 million doses of the vaccine were wasted due to delays in importations and poor cold chain management system. This wastage can pose a challenge to achieving COVID‐19 herd immunity in Nigeria. The causative factors of the vaccine wastage in Nigeria must therefore be tackled in order to effectively curb the COVID‐19 outbreak and consequently achieve herd immunity through massive vaccination. The government in collaboration with World Health Organisation and Africa Centre for Disease Control should facilitate and fast track the safe delivery of the vaccines to Nigeria. Misconceptions about COVID‐19 and its vaccine should be tackled to encourage the vaccine acceptance. The challenges faced by the vaccine supply chain system should be properly addressed by providing adequate funding and security, improved power supply, good vaccine infrastructure and improved vaccine data management in order to facilitate the safe and timely distribution of adequate vaccines across the country.

Dear Editor,

Africa has experienced a huge emblem of disease outbreaks such as the Coronavirus disease of 2019 (COVID‐19) that have endangered its public health‐care system in the twenty‐first century. The emergence of the COVID‐19 pandemic has impacted the socioeconomic and health‐care systems of many countries such as Nigeria and other African countries with fragile health systems. Since the emergence of the COVID‐19 in Nigeria, the novel pandemic has overburdened the country's public health system, and which is yet to recover from the pandemic's massive blow. As of 27 October 2022, Nigeria recorded 266,043 confirmed COVID‐19 cases, with 3155 deaths across the country [1], and the numbers keep growing on daily basis.

Many strategies such as temporary closures of border, internal travel restrictions, school closures, closure of worship places such as mosques and churches, physical distancing, mandatory use of face masks in public places and restrictions on mass gatherings such as conferences and wedding events have been put in place to curb the COVID‐19 pandemic in Nigeria and other countries. But vaccination remains the most effective strategy, particularly in developing countries such as Nigeria where most disease outbreak prevention measures have been poorly strategised or implemented. This prompted the swift development of COVID‐19 vaccine. Moreover, not long after COVID‐19 was declared a pandemic by the World Health Organisation (WHO) on 11 March 2020, a large quantity of safe and effective COVID‐19 vaccines were made available globally.

Nigeria was able to secure some doses of the vaccine such as; AstraZeneca, Moderna, Johnson & Johnson and Pfizer [2] through donations from countries with developed economies such as the United Kingdom, Canada and the United States of America. The donation was received from the COVAX facility and the African Union. The doses were made freely available in public and some designated private health facilities and COVID‐19 diagnostic centres across the country.

Despite the free availability of the COVID‐19 vaccine in Nigeria, it is worth noting that serious concerns have been raised as slews of the doses are being wasted in the country. In 2021, it was estimated that up to 1 million COVID‐19 vaccines have expired in Nigeria without being used [3] and the number keeps rising on daily basis. Nigeria's vaccine wastage seems to be one of the largest of its kind, outweighing the total number of vaccines received by some other African countries [3]. Most of the vaccines were wasted in Nigeria due to their short shelf‐life coupled with delays in importation as many doses expired because they were imported few weeks near expiration [3]. The low acceptance rate of the vaccine is also a contributing factor. Nigerians have been hesitant towards accepting the COVID‐19 vaccine as a result of concerns over its safety, side effects effectiveness and fear of the unknown. Many of the citizens also believe that COVID‐19 does not exist in Nigeria, and that its vaccination campaign is a means devised by some government officials to siphon public funds. However, the fragile nature of the vaccine supply chain system in Nigeria could be the arch cause of the massive vaccine wastage in the country. The supply chain management system is faced with many challenges such as underfunding, shortages of staff, inadequate and poor storage infrastructure, unstable power supply and frequent breakdown of vehicles [4]. These challenges hinder attaining recommended vaccine storage temperatures which greatly accelerate vaccine thawing and in turn, result in loss of potency [4], leaving the country with no option than to destroy the vaccine. Distribution challenges, poor vaccine data management, insecurity and weak or poorly implemented policies are other factors that cause the vaccine wastage in Nigeria [5]. The bad road networks especially in Nigeria's rural areas make it challenging for safe and timely vaccines distribution to target facilities [4]. The challenges faced by Nigeria's security system have also contributed to the delay in the COVID‐19 vaccine distributions and uptake [3, 5]. Banditry, the Boko‐Haram insurgency, cattle rustling, communal clashes and the rampant kidnappings across the country might have hindered the free movement of health workers, logistics officers and programme managers from accessing the rural facilities for fear of their health and safety, which could result to many COVID‐19 vaccine doses being unused. It is worth noting that the emergence of the COVID‐19 pandemic may have further complicated some of the challenges associated with medicines and vaccines supply chain in Nigeria [5].

Noting that Nigeria is the most populous country in Africa, with over 200 million population, the COVID‐19 vaccine wastage can be a challenge for the country towards controlling the COVID‐19 outbreak. It can disrupt the country's efforts towards achieving herd immunity against COVID‐19, as so far, less than 20% of its population are vaccinated against the virus [6]. Therefore, stringent measures should be devised in order to overcome the challenge. The government in collaboration with WHO and Africa Centre for Disease Control should facilitate and fast track the safe delivery of the vaccines to Nigeria and other African countries. Delivery of vaccines that are near expiration into Nigeria should be avoided. Misconceptions about COVID‐19 and its vaccine should be tackled through information dissemination, health education and promotion to encourage the vaccine acceptance among the citizens. This can be achieved by engaging pertinent stakeholders such as community leaders, civil society organisations, religious leaders, the mass media and community based organisations. Policymakers should ensure that vaccination policies are improved or fully implemented and adhered to. There is need for improved security and provision of adequate manpower in order to increase access to the vaccines in Nigeria, particularly in the rural areas that are heavily affected by insecurity. Infrastructural development and regular availability of electricity supply are keys to the success of the supply chain system for medicines and vaccines in the country. There is also a need for effective strengthening of the system through adequate budgetary provision. Producing its own vaccine should be a priority for Nigeria. This can be achieved by scaling up the local pharmaceutical industries and through collaborations with international pharmaceutical firms.

## AUTHORS CONTRIBUTION

Shuaibu Saidu Musa conceived the idea. Shuaibu Saidu Musa, Adamu Muhammad Ibrahim, Muhammad Rahima Hamid, Hope Jonah Obia and Garba Mohammed Buwa analysed the data and information and rotated in writing different versions of the drafts. All authors read and approved the final manuscript.

## CONFLICT OF INTEREST

Shuaibu Saidu Musa is a member of the Youth Editorial Board of Public Health Challenges. He was excluded from editorial decision‐making related to the acceptance of this article for publication in the journal.

## FUNDING INFORMATION

This research did not receive any specific grant from funding agencies in the public, commercial or not‐for‐profit sectors.
